# M. Andral, Jun. on Peritoneal Inflammation, Chronic and Acute. From the Hospital Practice of La Charite

**Published:** 1828-01-01

**Authors:** 


### PERITONEAL INFLAMMATION.

*Clinique Medicale.* Par G. ANDRAL, Fils.

[Hôpital de La Charité.]

IN a former number, (No. 11, for Jan. 1827, page 145—161,) we made our readers acquainted with a very able paper by M. Andral, on diseased conditions of the mucous membrane, and muscular coat of the stomach. We now proceed to the pathology of the peritoneum, occupying a considerable portion of the fourth volume of this able pathologist. The diseases of the liver will form a separate article, which we hope to include in our next number.

We have often drawn the attention of our brethren in this country to the pathological investigations of the French physicians, while we lamented the want of means—as well, perhaps, as the want of zeal, in these islands, which has so long and so materially thrown us into the rear in this particular branch of the science of disease. We every day hear it urged, in excuse, that the investigation of diseased structures leads men astray, and makes them mistake effects for causes. Granting that this is the case, it is only the abuse of a good thing. Is there any possible way of connecting with their appropriate symptoms, the origin, progress, and acmé of organic changes in the living body, but by numerous dissections performed at all periods of the disease, from the first disturbance of function to the ultimate annihilation of the same, by structural alteration? We say there cannot be any other mode of acquiring this important information. The ancients were, no doubt, very clever and very correct in noting down the symptoms of diseases, and observing their causes. But they could not possibly have formed correct notions of the conditions of internal organs, of which the symptoms were mere indications;—because they did not examine into these pathological conditions. Their diagnoses were, therefore, mere guess-work, and not founded on accurate data. They must have been much more frequently wrong in their diagnoses than modern pathologists. Let us not then attempt to separate these two branches of medical knowledge—symptomatology and morbid anatomy—for on their perpetual junction and mutual co-operation our prognosis and therapeia must depend. The Frenchman may lean too much on post-mortem investigations—the Englishman may trust too much to the observance of symptoms during life—but the wise man will endeavour to make each process throw light on the other. With these few preliminary remarks tending to bespeak the attention—perhaps, the patience of our readers, we shall proceed to give some account of M. Andral's researches on peritoneal inflammation.

### I. Acute Peritonitis.

In the following cases, our talented and indefatigable author wishes to draw attention, *first*, to certain causes which are very generally operative in the production of peritonitis—*secondly*, to the different symptoms which indicate the existence of this serious affection—*thirdly*, to the march of the disease, which, in some cases, is so rapid that only a few hours intervene between the origin of the inflammation and death; while, in other cases, the inflammation, though always acute, will not prove fatal till after the lapse of thirty or forty days.

ILLUSTRATIONS.

*Case* 1. A boy, 15 years of age, of feeble constitution, a compositor in a printing office, went to work, in his usual health, on the morning of the 30th April. At 2, P. M. he felt a pain in the lower part of the right side of the abdomen, which obliged him to break off from his work. In the course of the night, the pain extended to the hypochondriac and epigastric regions, accompanied by vomiting and great prostration of strength. These symptoms continued the next two days, during which, he lay in bed and drank diluent drinks. On the 2d May, he presented the following symptoms:—face pale, and expressive of great anxiety—eyes dull—sensorial functions undisturbed—abdomen tense, and exquisitely painful on pressure, especially in the right side—frequent vomiting of bilious matters—obstinate constipation—tongue moist and white—pulse rather quick—skin hot and dry. *Venesection to* 12 *ounces,* 30 *leeches to the abdomen—fomentations—lavements.* The blood was inflamed. The pain was somewhat relieved. *May 3d.* Twenty leeches were applied to the abdomen. The vomiting ceased this day. *4th.* All the symptoms were ameliorated; but the abdominal tension continued—hence it was inferred that the inflammation had passed into a chronic state. Simple *ptisans—fomentations—low diet.* That evening he received cold from a window which had been left open, and next day, he was found in articulo mortis, when the physician went round.

*Dissection.* There was no appreciable lesion of the cerebro-spinal system, nor in the viscera of the thorax. In the abdomen, the peritoneal covering of the small intestines was remarkably injected, and a considerable effusion of whitish fluid in the hollow of the right ilium and in the pelvis. The stomach, liver, and colon, were covered with white concretions of a membraniform appearance. The mucous membrane of the stomach was pale. The same might be said of the small intestines, except a portion of ileum, near the ileo-cæcal valve, where the lining membrane was injected.

*Remarks.* The above case presents one of the finest specimens of uncomplicated peritonitis that can well be imagined, accompanied by well-defined symptoms. Here there was no apparent prodrome, or, as Dr. Marsh would say, "latent period," between sound health and violent disease. Pain was the first symptom, at first partial, and then more extended, with vomiting, and alteration of the features—all very characteristic of this dangerous kind of inflammation. The pulse here, as in many other cases, was deceptive, and its action not at all in proportion to the dangerous disease that was going on. The following case presents some curious traits.

*Case* 2. A young man, 18 years of age, had enjoyed habitual good health, till the 2d March, when, without any apparent cause, he was seized with sharp pains in the abdomen, not constant, nor always in the same place. He kept to his room for five days, but took no medicine. On the seventh day of his illness, he came into La Charité, presenting the following symptoms:—face flushed—abdomen distended and tense, without any fluctuation—great sensibility to pressure—pulse small, very quick, and rather irregular—skin hot and dry—tongue coated yellow—constipation. Notwithstanding the time that had elapsed since the invasion of the disease, depletion was determined on, and 30 leeches were applied to the abdomen, to which were added lavements and fomentations. The patient was greatly relieved, the abdomen becoming less tense and painful. Leeches were again applied. For three days the sickness at stomach ceased—and a diarrhœa came on. Still the tension and tenderness of abdomen evinced that peritoneal inflammation was going on. By the 12th day of the disease, the abdomen was considerably distended, and very painful. The hypogastric region was covered with leeches, which reduced the swelling and pain. On the 14th day, the patient demanded his discharge from the hospital, as they did not give him enough of food. He got up and dressed himself, but was unable to leave the institution. The next morning he suddenly expired, being the fifteenth day from the commencement of the symptoms.

*Dissection.* The peritoneum was adherent to the convolutions of the bowels, by white and thick layers of recent formation. Beneath these the membrane itself was highly injected. There was considerable milky effusion in the pelvis. The internal surface of the stomach was pale. The same with the intestines. There was no other appreciable lesion in the body.

*Remarks.* The above is another case of pure peritonitis, and is remarkable for the craving of food, and the suddenness of the death. These two cases prove, as do thousands of others, that the different tissues may be separately inflamed, though such doctrine is very much scouted by our high-bred routine physicians, who dislike the trouble of accurate investigation and close observation.

*Case 3. Peritoneal Inflammation succeeding Rheumatism.* In a former volume, our indefatigable pathologist traced several cases of pleuritis, pulmonitis, and pericarditis, to sudden cessations of acute rheumatism. He observes that it is of little use to cavil about the term *metastasis*, in such cases, provided we bear in mind the fact, that a *sudden* disappearance of articular inflammation is occasionally followed by phlegmasia of an internal organ—and especially the serous tissues of those organs.

*Example.* A man, 57 years of age, was received into La Charité labouring under acute rheumatism, which shifted from joint to joint. *Several venesections* were practised. One day, the rheumatism suddenly ceased in the articulations then affected, and did not assail any of the others. But acute pains were soon felt in the abdomen, which became so violent, that the patient was forced to send forth the most piercing cies. This appeared like *metastasis*, and sinapisms were applied to the articulations originally inflamed, while leeches in great numbers were clustered on the abdomen. Ultimately the patient was put into a warm bath. These means had no good effect—the pain invaded all parts of the abdomen, which enlarged much, and presented fluctuation. Death took place on the third day from the recession of the rheumatism.

*Dissection.* As soon as the abdomen was opened a flood of reddish fluid issued forth, containing some floating flocculi. The intestines were intensely red, and adhesions were forming in various places. The effused fluid resembled venous blood, but there were no coagula. There was no affection of the mucous membrane of stomach or bowels.

A curious formation was discovered in the examination of this man. From the fundus of the bladder there went off an oval pouch, which reached and adhered to the duodenum. It communicated with the main body of the bladder by an opening resembling the pylorus. The structure of this pouch or appendix was, in all respects, similar to that of the bladder itself.

We think the most sceptical pathologist will hardly deny that there was here a transference of inflammation from the joints to the peritoneum. We observe that the patient was bled *several times* for acute rheumatism. We are daily astonished and grieved to see the want of discrimination in bleeding for acute rheumatism. The generality of medical men are now getting it into their heads that inflammation is inflammation, wherever seated—however designated; and, consequently, that there is but one class of remedies—venesection and decisive depletion. It is no wonder that, under such routine practice, we see so many examples of metastasis of inflammation to the heart. Another measure which is very generally had recourse to, in acute rheumatism, is the warm, or rather the hot bath. This is a most dangerous procedure. It determines the tide of the circulation strongly to the surface, no doubt—but there is a subsequent retreat of that tide, which often sweeps with it the inflammation from the joints to the interior. Of this we have seen examples which left no doubt on our minds as to the fact.

*Case* 4. A curious pathological fact has, in these days of diligent investigation, been pretty fairly established—namely, that irritation or inflammation in the mucous membrane of the duodenum will sometimes produce jaundice, where no obstruction can be detected in the biliary ducts. This fact, we think, will ultimately throw some light on the nature of yellow fever. The following is an instance of this kind.

A female was confined, and the delivery was followed by profuse hæmorrhage. This was combated by cold applied to the hypogastrium, and by the introduction of *lemon juice into the cervix uteri.* On the fourth day, the lochia became suppressed, and the abdomen became the seat of severe pain. She was admitted into La Charité, her belly immensely distended and tympanitic, and accompanied by fever of the puerperal kind. Leeches and the usual means were employed, but without advantage. Next day she became completely jaundiced—and on the 3d day of the present illness she died.

*Dissection.* Great quantities of gas were confined in the intestines—these last were covered with albuminous effusions—and whitish puriform secretions were collected in the pelvis. The mucous membrane of the stomach was pale; but that of the duodenum was inflamed. On minute examination, no affection or obstruction of the liver or its ducts could be detected. The internal surface of the uterus was inflamed.

Dr. Andral thinks that the tympanitis may have occasioned the peritonitis. But extrication and accumulation of air in the bowels are such usual attendants on peritoneal inflammation, that we can hardly regard it in any other light than as the *effect* of the inflammation.

Here we must quit the subject of *acute* peritonitis. The symptoms of this dangerous disease are nearly as unequivocal as those of any other inflammation, though the treatment is more difficult. It is chronic inflammation of the peritoneum which produces such havoc, and which so generally passes undetected till it is too late for remedy. There are many cases of chronic peritonitis, where the disease goes on to fatal effusion—to tuberculation—or to adhesion of the intestines into a mass, and yet no *pain* may have been complained of at any period of the disease. More generally, however, chronic, is a sequel of acute peritonitis. We shall be able to introduce but two or three cases, offering peculiar features, before we close this article.

*Case* 5. A tailor, 24 years of age, was seized with abdominal pains, in the beginning of December, attended with diarrhœa. He kept his room for three weeks, and then came into La Charité. The abdomen was distended, and fluctuation was obscurely perceptible. The diarrhœa continued—the tongue was red at the point—vomiting—quick pulse—cough. Auscultation and percussion could detect no disease of the lungs; but they had no doubt of inflammation, both of the peritoneum and mucous membrane of the bowels. *Leeches, fomentations, low diet.* During the next four or five days there was no diarrhœa, but the symptoms of peritonitis continued. During the remainder of January, the abdomen got larger, the pulse very quick, and the skin dry and rather hot. In the beginning of February, the cough increased, and some oppression was felt. The chest, however, sounded well, and the respiration was heard throughout, without any wheeze. He died, exhausted, on the 15th February.

*Dissection.* The abdominal parietes were strongly adherent to the intestines—and there was an effusion into the abdomen, of a brown colour and fæcal odour. The small intestines were glued together, and covered with false membranes, which membranes were studded with tubercles. Beneath these membranes, the peritoneum was found of its natural colour and structure. Between the peritoneal and mucous membranes, a number of tubercles were developed, some of which were softened down, and had burst through the peritoneal covering. Within a few inches of the ileo-cæcal valve, the coats of the ileum had given way, and there existed a perforation. The mucous membrane of stomach and bowels was pale and healthy. There were some crude tubercles at the summit of each lung—and the pericardium was adherent to the heart by a thick layer of false membrane, studded with tubercles.

*Remarks.* The above is a very well marked case of tuberculated accretion of the serous membranes, with effusion—all, no doubt, the consequence of chronic inflammation. This case also presents a specimen of perforation of the intestine, proceeding *from without inwards,* and caused by the softening down of tubercles. The following is another striking example of the ravages which chronic peritonitis is capable of effecting before death.

*Case* 6. A shoemaker, aged 19 years, experienced, in the month of May, some acute pain in the abdomen, which did not, however, prevent him from work for some days. At last he took to his bed. There was pain on pressure, but no vomiting or diarrhœa. There was cough and fever every evening. In July, there came on a diarrhœa, and, on the 12th August, he entered LA CHARITÉ. His face was pallid and swelled—some œdema of the ankles—pain about the umbilicus augmented by pressure—belly rather tumid and presenting fluctuation—three or four liquid stools daily—slight cough—quick pulse—morning perspirations—great emaciation of the thoracic parietes and the arms. In the course of the month of August, a strict regimen and fomentations entirely relieved the pain; but the cough increased—the diarrhœa continued—the perspirations became more profuse—the debility and marasmus made rapid advances, and the patient died on the 31st of the same month.

*Dissection.* There were several ounces of serum in the pericardium, the heart itself being apparently sound—superior lobe of the left lung converted into a tuberculated mass, leaving scarcely a trace of parenchymatous structure. Numerous tubercles in the rest of the lungs. In the abdomen, a considerable quantity of yellow serum—epiploon greatly indurated and tuberculated—intestines glued together by false membranes, these membranes being studded with tubercles—internal surface of the stomach pale, except near the pylorus, where it was discoloured —an ulceration close to the ileo-cæcal valve above—and one or two below that apparatus. The valve itself was ulcerated, and a complete *perforation* of the coats effected.

The above case offers an instance, to which the attentive practitioner would often find parallels, where ulceration of the intestines takes place, attended with so little pain, that the disease would not be at all suspected. We believe, indeed, that, in the present state of our knowledge, there is really no pathognomonic sign by which we can ascertain the existence of this dangerous—generally fatal malady! This uncertainty, however, should put us on our guard against that system of drastic purgation, now so indiscriminately put in practice by *routinists*, without thought or consideration.

Here our review of M. Andral's work must terminate, for the present. The work abounds with ample illustrations of abdominal inflammation, drawn from the clinical practice of a public hospital. They are highly deserving of attention. We are gratified to observe that, in all our medical discussions, in the various societies of this metropolis, the subject of pathology, as cultivated by our continental neighbours, is now exciting universal interest, and that what we have long urged in this Journal, is confessed with one voice—the superiority of continental pathology over that of this country. It is humiliating to observe the sneers with which our fashionable physicians, in this metropolis, regard the study of pathology. Dr. Hodgkin, in his paper on Medical Education, lately read and discussed at Guy's Hospital, alluded to the disgraceful fact, that—in the theatre of the Royal College of Physicians of London, a systematic attempt was made to depreciate the study of morbid anatomy! Such a fact will place us in a pretty light on the Continent! But we trust the present, and especially the rising generation, will wipe off this foul disgrace to science, and prove that Englishmen, when once aroused, will evince that native energy so strongly inherent in them, and shew that they are not to be outdone in any pursuit, where the health and comfort of their countrymen are concerned.

